# Introducing an MDT Morning Huddle on an Adult Inpatient Unit During the COVID-19 Pandemic

**DOI:** 10.1192/bjo.2022.326

**Published:** 2022-06-20

**Authors:** Sita Parmar, Tomasz Pierscionek, Helen Souchon, Naomi Thompson, Amina Shahzad, Rithika Koshy, Nokuthula Marks, Victor Doku, Saraspadee Veeramah

**Affiliations:** 1 South London and Maudsley NHS Foundation Trust, London, United Kingdom; 2 Oxleas NHS Foundation Trust, London, United Kingdom; 3 Croydon Health Services NHS Trust, London, United Kingdom; 4 Imperial College Healthcare NHS Trust, London, United Kingdom

## Abstract

**Aims:**

This quality improvement project aimed to introduce a staff communication tool, ‘the morning huddle’, to Goddington ward (Green Parks House, Oxleas NHS Foundation Trust). The huddle's purpose was to share key information between members of the ward's multidisciplinary team (MDT) regarding patient risk, diagnosis, mental state and treatment progress.

**Methods:**

The project team worked with the Oxleas Quality Improvement (QI) team to create a structured huddle with agreed goals that commenced at 9am each morning. The team sought views from ward staff on the existing communication process before implementing the huddle via a series of weekly questionnaires. The morning huddle was introduced on 26th May 2020 and all members of the team were invited (including the ward consultant, junior doctors, ward manager, nursing staff, healthcare assistants, psychologists, occupational therapist, and ward administrator).

Following multiple PDSA (Plan, Do, Study, Act) cycles, the team further refined the morning huddle into a meeting with a set template that included COVID-19 test results, psychiatric risk concerns, medication adherence, and barriers to discharge. The project team also timed the huddle, aiming for it to last a maximum of 30 minutes. A questionnaire was distributed to ward staff weekly after the huddle was implemented to ascertain their views on the process. Data collection for the project ended on 2nd August 2020.

**Results:**

The project's main outcomes were based on two questions from the weekly staff questionnaire:

1. “How effective did staff members find the morning huddle in addressing their concerns about patients and promoting safety of patients and staff?”

This improved over the course of the project, starting with 20% of staff finding the huddle “good” or “very good” in its effectiveness to 77% finding it “good” or “very good” in the final questionnaire.

2. “How effectively do staff feel that their concerns about patients are addressed by the rest of the team?”

This also improved, starting with 40% of staff selecting “well” or “very well” to 100% in the final questionnaire.

**Conclusion:**

Goddington ward introduced a huddle that was valued by the entire MDT. The huddle improved how well staff felt their concerns about patients were addressed and a noticeable improvement in team morale was observed. While the project succeeded in implementing a huddle that staff appreciated, patient outcomes also need to be considered in future

